# Deformation of Single Crystals, Polycrystalline Materials, and Thin Films: A Review

**DOI:** 10.3390/ma12122003

**Published:** 2019-06-22

**Authors:** Guijun Yang, Soo-Jin Park

**Affiliations:** Department of Chemistry, Inha University, 100 Inharo, Incheon 22212, Korea; yanggj91@gmail.com

**Keywords:** single crystal, polycrystalline material, thin film, deformation, mechanism, model

## Abstract

With the rapid development of nano-preparation processes, nanocrystalline materials have been widely developed in the fields of mechanics, electricity, optics, and thermal physics. Compared to the case of coarse-grained or amorphous materials, plastic deformation in nanomaterials is limited by the reduction in feature size, so that they generally have high strength, but the toughness is relatively high. The “reciprocal relationship” between the strength and toughness of nanomaterials limits the large-scale application and development of nanomaterials. Therefore, the maintenance of high toughness while improving the strength of nanomaterials is an urgent problem to be solved. So far, although the relevant mechanism affecting the deformation of nanocrystalline materials has made a big breakthrough, it is still not very clear. Therefore, this paper introduces the basic deformation type, mechanism, and model of single crystals, polycrystalline materials, and thin films, and aims to provide literature support for future research.

## 1. Introduction

In recent years, nanostructured materials have become a rapidly developing multidisciplinary research field. In the mid-1980s, Gleiter et al. [1] first proposed the concept of nanostructured materials, marking the era of research on these materials. A nanostructured material refers to a solid material composed of crystalline or amorphous ultrafine particles of the order of 1–100 nm. A single crystal refers to a solid state in which molecules (atoms or ions) contained in the sample are regularly and periodically arranged in a three-dimensional space. Polycrystals are composed of many small grains that have the same arrangement but are inconsistent in orientation. The nanostructured material contains a large number of grain boundaries or other interfaces, and has extremely high interfacial strengthening ability, thus exhibiting distinctive mechanical properties. It is represented by the Hall–Petch Equation (1) [2]:

(1) $$\sigma_{Y}=\sigma_{O}+kd^{-1/2}$$

where *σ_Y_* is the yield strength, *σ*_0_ is the lattice resistance, *k* is the material correlation coefficient, and d is the average grain size. This is an approximation. The more general formula is to use a power expression with exponent n, where 0.3 ≤ n ≤ 0.7. However, the Hall–Petch equation is strictly valid for polycrystalline materials with grain size larger than 1 μm, whereas for nanocrystals there are some significant deviations. When the metal grain size is reduced to the nanometer scale (*d* < 100 nm), the strength of the material will be significantly improved [3]. Nanocrystalline materials have extremely high yield strength but extremely low toughness. The coarse crystals have very high toughness but they sacrifice strength. To achieve high yield strength and good plastic matching, it is necessary to improve the work-hardening ability of the material. 

Generally, nanostructured materials mainly include atomic clusters, layered (lamellar) films, filamentary structures, and bulk nanostructured materials [4]. Nanomaterials have a large specific surface area, and the number of surface atoms, surface energy, and surface tension increase with a decrease in particle size. The small size effect, surface effect, quantum size effect, and macroscopic quantum tunneling effect lead to nanocrystalline materials having excellent physical, mechanical, and chemical properties, which leads to the wide application of nanomaterials in mechanics, electricity, optics, and thermal physics. In many cases, atomic and molecular interactions in the nanoscale range strongly influence the macroscopic nature of the material. Therefore, the physical properties of the nanomaterial may be quite different from the macroscopic properties of the same material. For example, Pachlaa et al. [5] used a hydrostatic extrusion process to refine the grain of commercial pure titanium (~33 μm). The obtained nano-titanium has a particle size of ~47 nm and exhibits an extremely high ultimate tensile strength (> 1300 MPa) and yield stress (~1250 MPa). In the field of ceramics, the toughness and strength of ceramics increase significantly after grain refinement treatment [6]. Strength and toughness (or plasticity) as the core mechanical properties of metals determine their performance in engineering applications. Strength is a stress that represents the resistance of a material to irreversible deformation, whereas toughness is the energy absorbed by a unit per unit volume before breaking. Obtaining better toughness while maintaining a certain strength is an important requirement for most structural materials. However, these attributes are usually mutually exclusive [7]. Studies have shown that the strength and toughness of nanostructured metal materials prepared by traditional strengthening methods are generally inversely proportional, that is, when the grain size changes from micron to nanoscale, the strength and hardness of the metal crystal are significantly improved. However, the material also becomes prone to brittle fracture. The microscopic plastic deformation mechanism is attributed to the fact that a large number of grain boundaries and second-phase inclusions introduced by conventional strengthening methods (such as grain boundary strengthening and dispersion strengthening) inhibit the movement of lattice dislocations. Twinning and slip are the basic plastic deformation mechanisms of polycrystalline materials. Although defects can hinder lattice dislocations and increase material strength, they also reduce the ability of materials to flow plastically. This phenomenon severely limits the applicability of polycrystalline materials. Many researchers have found that some nanocrystalline materials exhibit good tensile ductility and toughness [8,9,10,11,12] at a wide range of temperatures. The unique structural characteristics and phenomena of these materials accord them high strength and good ductility, which has caused widespread concern. Until now, many models have been proposed to explain this phenomenon. The high resistance of some nanocrystalline materials to fracture is mostly attributed to special deformation modes, such as lattice dislocation slip [13,14], cross slip [15,16], coble creep [17,18], rotational deformation [19,20], grain boundary slip [21,22], grain boundary migration [23,24], and nanoscale twin deformation [25,26]. These special modes of plastic deformation not only release high stress concentrations but also hinder the nucleation of nanocracks; they can also slow down or organize the expansion of existing cracks. However, it is not easy to correctly describe the deformation mechanism of nanostructured materials. To understand the basic principles of the excellent mechanical properties of nanocrystalline materials, it is necessary to explore the deformation mechanism of nanostructured materials. 

This paper briefly reviews the development and deformation mechanisms of nanostructured materials, focuses on the analysis and discussion of the effects of various nanostructure defects on the microstructure evolution and strengthening mechanism of materials, and improves readability into the multiscale methods of deformation mechanisms of nanomaterials and thin-film materials.

## 2. Deformation of Materials

**Figure 1:**
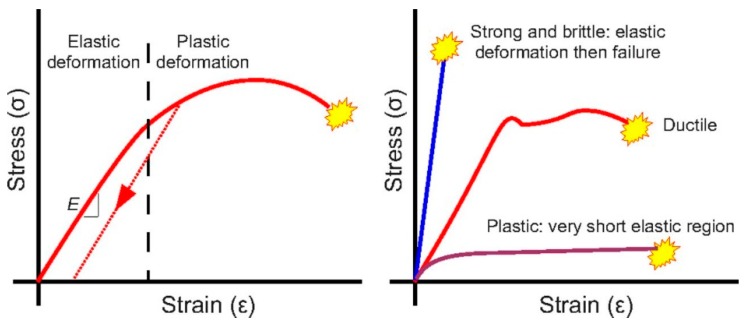
Stress/strain curves. **Left panel**: a classical curve, showing elastic and plastic regions, with hysteresis (dashed line); one deformation enters the plastic range—the material does not regain the original shape when the stressor is removed. **Right panel**: stress–strain curves for a variety of different materials (and possibly organisms).

The deformation of materials is divided into two types—elastic deformation and plastic deformation [27]. Figure 1 shows a typical stress–strain curves [28]. Plastic deformation of a substance (including fluids and solids) is caused by the action of an external force under certain conditions. Elastic deformation can be restored to its original shape or size after the external force is removed. After the external force is removed, the elastic deformation part disappears, and the part of the deformation that cannot be recovered, and remains, is called plastic deformation. When the material is stressed, the relative positions of the atoms change. When local deformation exceeds a certain limit, the bonding force between the atoms is destroyed, causing cracks to occur. These cracks expand to break the material. The main difference between elastic deformation and plastic deformation is that elastic deformation is reversible, and plastic deformation is irreversible.

### 2.1. Deformation of Single Crystals

#### 2.1.1. Deformation Types in Single Crystals

**Figure 2:**
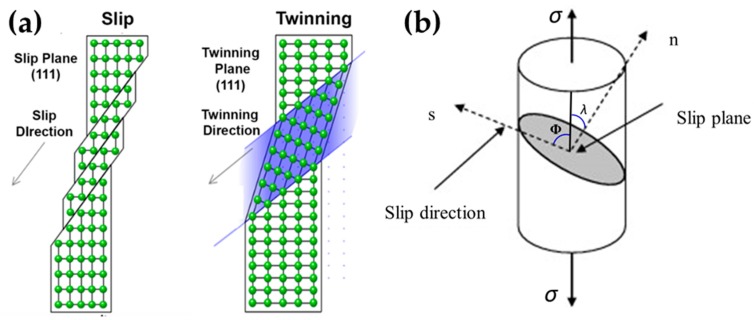
(**a**) Slip and twinning deformations in a face-centered cubic crystal. (Left) Slip occurs when dislocations glide through the material, causing crystal planes to slide along each other. (Right) Twinning occurs via a concerted shift of atomic positions in the twinning direction. The twin crystal’s structure is a mirror image of the parent structure about the twinning plane. (**b**) Where n is the normal vector to the slip plane and s is the slip direction.

In general, the plastic deformation of a single crystal can be divided into two types: slip and twinning (Figure 2a) [29]. Slip refers to sliding of a part of the crystal on a certain crystal plane (slip plane) along a certain direction (slip direction) relative to another part under the action of shear stress. The combination of the slip surface and the slip direction is called the slip system. Slip is more likely to occur in some crystal directions and planes than in other directions. Slip is caused by dislocation motion. To move the dislocations, certain stresses must be applied to overcome the resistance of the dislocation motion. Slip occurs when the shear stress applied to the slip direction on the slip surface reaches a certain critical value.

This critical resolved shear stress (CRSS) is related to the stress required to move the dislocations on the slip plane. Schmid’s law states that when the shear stress acting along the slip direction on the slip plane reaches a critical value τ_c_, crystal dislocation occurs (Figure 2b) [30]:

(2) $$\tau_{c}=\sigma \;\cos\Phi \cos\lambda$$

In this formula, τ_c_ is the critical resolved shear stress, σ is the tensile stress, φ is the angle between the slip direction and the direction of the applied force, λ is the angle between the normal of the slip plane and the direction of the applied force, and cosφcosλ is the Schmid factor (M). Where n is the normal vector to the slip plane and s is the slip direction. When λ = φ = 45° (soft orientation), τ = σ/2, the slip system possess the largest tensile stress; when λ = 90° or φ = 90° (hard orientation), τ = 0. Crystal orientation can make the movement of dislocations easy or difficult. Under identical conditions, more the slip systems in the crystal, more will be the spatial orientations that can be assumed during the slip process, which is beneficial for slip and, therefore, leads to high plasticity.

The common face-centered cubic (FCC) crystal has a slip direction of <1 1 0> and a slip plane of {1 1 1}. Therefore, the face-centered cubic crystal has 12 slip systems. The slip direction of the body-centered cubic (BCC) crystal is <1 1 1> and the slip surfaces that may appear are {1 1 0}, {1 1 2}, {1 2 3}. Thus, there are 48 potential slip systems for the BCC crystal. Although there are more slip systems in the BCC crystal than in the FCC crystal, the slip systems of BCC cannot appear simultaneously. Therefore, the plasticity of the BCC crystal is not as good as that of the FCC crystal. The slip direction of the hexagonal close-packed (HCP) crystal is <1, 1, −2, 0>; the slip surface is {0, 0, 0, 1}, the prism surface is {1, 0, −1, 0} and the pyramidal surface is {1, 0, −1, 1}. There are three slip systems of HCP. Owing to the small number of slip systems of HCP, its plasticity is generally not as good as FCC and BCC [31,32]. Table 1 shows the critical resolved shear stress of some metal single crystals with different crystal structures at room temperature [33]. As can be seen from the table, impurities can affect the CRSS of the material significantly. Srinivasan et al. [34] reported the effect of impurities on the CRSS of single crystals, which was investigated by compressive experiments on magnesium oxide single crystals containing 40 mol ppm Fe^3+^ and 500 mol ppm Ni^2+^ impurities. In a pure crystal containing a small amount of Fe^2+^, two different strain-hardening regions were identified with a CRSS of 0.73 ± 0.07 kg/mm^2^. Moreover, even if the concentration of Fe^3+^ is very low, precipitation-hardening can be observed during aging. The effect of heat treatment on Ni^+2^-doped crystals is very small. Studies have shown that Fe^3+^ has a much greater effect on MgO hardening than Ni^2+^. 

In addition to impurities, many factors affect the CRSS of materials. Spitzig et al. [35] studied the effect of orientation and temperature on the plastic flow properties of iron single crystals. The results show that with a decrease in temperature, the increase in BCC CRSS is significantly larger than that of FCC CRSS. In addition, the slip of the BCC crystal and its dependence on crystal orientation and temperature are much more complicated than those of the FCC crystal. Wu et al. [36] studied the effects of particle size and orientation on single-crystal aluminum, compared the results with other previously reported data on FCC crystals (Ni, Cu, and Ag), and established a sample size effect trend on superimposed fault energy. The orientation dependence of Al single crystals maintains good agreement with the Schmid factor relationship and the extracted critical resolution shear stress even at the micron scale and nanoscale. Moreover, higher the energy of the stacking fault, higher will be the dislocation mobility and lower will be the influence of the sample size. 

Twinning is another type of plastic deformation that occurs when it is not possible for the crystal to slip. Under the action of shear stress, some parallel crystal planes in a local area of the crystal produce uniform shearing at a distance relative to each other along a certain direction. The deformed and undeformed two-part crystal is referred to as a twin crystal. The interface between the uniform shear region and the nonshear region is called the twin boundary and the moving direction of the twin plane becomes the twinning direction (Figure 2a) [29]. In the shear zone, the relative displacement of each layer of the crystal plane parallel to the twin plane is constant, which is common in HCP and BCC crystals. The twin deformation also occurs under the action of shear stress, usually in the stress concentration zone caused by the slip resistance. Therefore, the critical shear stress required for twinning is much larger than that for slip. Twinning is uniform shear compared to the uneven deformation caused by slip. Sliding directly produces plastic deformation, and twinning changes the crystal orientation, resulting in a new slip system, which has an indirect effect on plastic deformation. Yu et al. [37] reported the strong crystal size effect on deformation twinning by micro-compression and static compression experiments. It was found that the stress required for deformation twinning increases sharply with the decrease in sample size until the sample size is reduced to 1 micron. In this range, the deformation twins are completely correlated. The stress is replaced by a less common general dislocation plasticity. In some HCP metals, such as Mg, under the very low stress of *c*-axis stretching, there will be tension twinning or stretching twins. It has a significant contribution to structural evolution and a substantial effect on plastic flow during large deformations. Rawat et al. [38] used the molecular dynamics (MD) simulation method to study the evolution of {10$\bar{1}$2} twins in Mg single crystals with initial cracks. The effects of multiaxial loads on the evolution of different twin volume fractions and twin numbers were also reported. In this review, we compared five loading conditions. The results showed that all of the samples exhibited the characteristic of twin binding, and, in addition to the binding between the twins with the same variant, binding occurred between the twins belonging to the conjugated variant. The activation of twin mutations is consistent with the Schmid factor. However, variants with the same Schmid factor are not necessarily activated simultaneously. Furthermore, active twin variants with the same Schmid factor do not evolve at the same rate; thus, they contribute differently to the entire twin volume fraction. Narayanan et al. [39] studied the strain-hardening behavior and size effect of five-fold twinned Ag nanowires (AgNWs). Strain-hardening in five-fold double-crystal AgNWs does not give rise to single-crystal nanowires or other nanostructures. The smaller wire is significantly hardened and its ultimate tensile strength is higher than that of the larger Ag wire. In addition, surface toughness [40], impurities [41,42], or orientations [43,44] can have a significant effect on twin deformation.

#### 2.1.2. Deformation Mechanism in Single Crystals

The plastic deformation of crystals is a complicated process and it is of significance to study the deformation mechanism. Yoshinaga et al. [45] studied the deformation mechanism of magnesium using a single-crystal compression strain method parallel to the hexagonal axis. These deformation mechanisms were found to be twins along different composition planes, including the ribbon axes of <11$\bar{2}$0> or <10$\bar{1}$0>, but no sliding deformation effective for such compression was observed under the optical microscope. The CRSSs of these two structures were determined to be very high (approximately twice the non-base slip) and they decreased rapidly as the test temperature increased.

**Figure 3:**
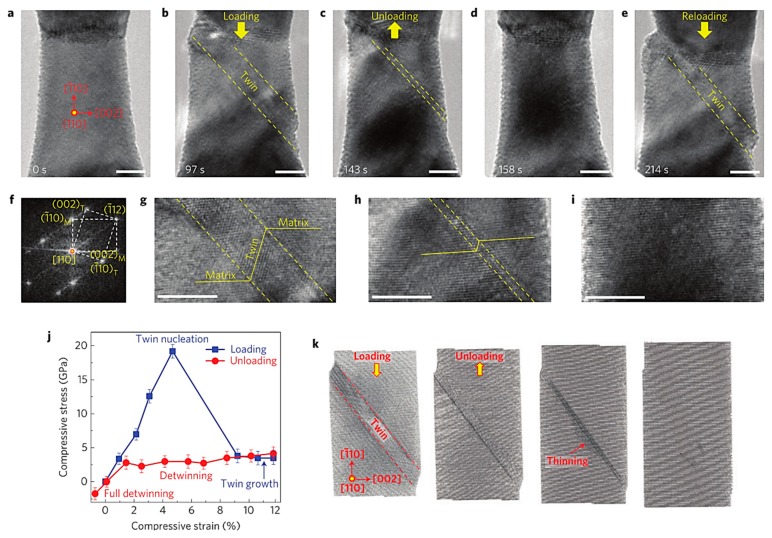
Reversible deformation twinning and detwinning processes in a W bicrystal nanowire under cyclic loading. (**a**) Pristine W bicrystal nanowire with a diameter of 14.7 nm as viewed along [110] and loaded along [$\bar{1}$10]. (**b**,**g**) Under compression, the deformation twin nucleates and expands to approximately 4 nm in thickness. (**c**,**h**) A layer-by-layer detwinning process occurs upon unloading. (**d**,**i**) After complete detwinning, the W bicrystal recovers its original shape (**e**). A deformation twin nucleates at the same place in subsequent deformation cycles. (**f**) Fast Fourier transform pattern of the deformation twin. (**j**) Stress versus strain curve during compressive loading and unloading, showing the pseudoelastic response. The stress is estimated based of the lattice strain, and the overall compressive strain is measured from the change of the nanowire length. The error bars represent the variations of the estimated stresses at different locations of the nanowire. (**k**) MD snapshots showing the twinning–detwinning process similar to TEM observations. All scale bars: 5 nm.

In general, low stacking faults can catalyze cracking metals and alloys with high twinning activity, whereas high stacking faults can catalyze slippage of cracked metals and alloys. Kim et al. [46] revealed the deformation and twinning mechanism of Fe-17Mn-0.45C-1.5Al-1Si TWIP (twinning-induced plasticity) steel. In situ transmission electron microscopy showed that the thin smear of the Shockley local dislocations formed at the grain boundaries indicates the nucleation process of the deformed twins at the grain boundary defects. The observed twinning process is similar to the deformation twinning process produced by the continuous emission of partial dislocations at the grain boundaries in the nanocrystalline material. In addition, high-density Frank partial dislocations were observed inside the deformed twins. These defects affect the growth of deformed twins and may result in high work-hardening of TWIP steel. Karaman et al. [47] studied the stress–strain behavior of Hardfield steel (Fe, 12.34 Mn, 1.03 C, wt%) single crystal under tension and compression. The overall stress–strain response is strongly dependent on the crystal orientation and applied stress direction. Studies have shown that twins are the main deformation mechanism in the compression of crystals at the beginning of crystal and inelastic deformation. The main deformation mechanism of crystal deformation and crystal tensile deformation is multislip. In addition, the crystal undergoes a single slip during stretching and compression, and planar dislocations occur. Wang et al. [48] studied the main deformation mechanism of BCC tungsten (W) nanocrystals using in situ high-resolution transmission electron microscopy (HR-TEM) and atomic simulation. The results show that the twin crystal is the main deformation mechanism of BCC tungsten nanocrystals. This deformed twin is pseudoelastic and exhibits reversible decrystallization during unloading. In addition, the synergy between twinning and dislocation slip can be adjusted by the loading orientation, which is related to the nucleation mechanism of nanoscale BCC crystal defects (Figure 3). Table 2 lists the largest Schmid factors on the dislocation slip and deformation twinning systems for the four loading orientations tested in BCC W nanowires. This work provides new insights into the deformation mechanism of BCC nanostructures.

### 2.2. Deformation of Polycrystals

The plastic deformation of each of the crystal grains in a polycrystal is substantially similar to that of a single crystal, and the polymorphous plastic shape is still slip-like. Kocks et al. [49] compared single crystals and polycrystals on the aspects of flow stress, work-hardening, temperature, strain rate effect, and grain size, and analyzed the relationship between single crystals and polycrystals in detail. The mechanism of plastic deformation is that dislocations occur in the crystal under the action of shear stress, sliding along a certain crystal plane to achieve plastic deformation of the metal. As the lattice orientation of each crystal grain in the polycrystal is different, the arrangement of atoms at the interface between the crystal grains is extremely irregular, and the grain deformation is both intragranular and intergranular. Thus, the plastic deformation of the polycrystal is smaller than that of the single crystal. When polycrystalline materials are plastically deformed, not all grains are slipped simultaneously—the crystal grains are staged and slipped in batches through the application of external force. The main mechanism is the slip inside each grain, accompanied by the slip and rotation between the grains. Owing to the unevenness of the deformation, various internal stresses are generated in the deformed body, which do not disappear after the deformation is completed and ultimately become residual stress. Residual stress can play a role in strengthening or weakening the working stress. If the residual stress is a large tensile stress, the metal will be destroyed under the condition of small load, and the residual tensile stress of the surface layer will promote the formation of fatigue cracks, which will lead to considerable damage to the parts under alternating load working conditions. However, if the existence of residual stress reduces the working tensile stress, the service life of the part can be improved, for example, by intentionally manufacturing compressive residual stress, shot peening, and nitriding on the surface of the work piece. 

**Figure 4:**
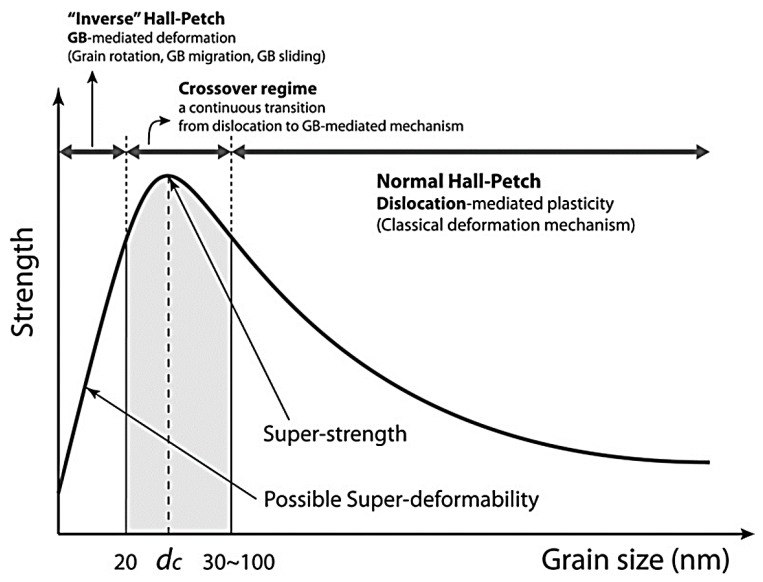
Strength of polycrystalline materials as a function of grain size: Hall–Petch relation (classical deformation mechanism) and transition to “inverse’’ Hall–Petch relation (grain sizes below 20 nm).

The mechanical properties of polycrystalline materials can be adjusted by varying the degree of nucleation, propagation, and interaction of dislocations and shear bands. As metals and alloys are mostly polymorphic, the interaction between defects and grain boundaries is particularly significant. The grain boundaries accord higher strength and hardness to polycrystals than that of single crystals. The finer the crystal grains in polycrystals, the larger the ratio of grain boundary regions and the strength and hardness of metals and alloys. In bulk polycrystalline materials, the grain boundary acts as a barrier to dislocation motion, expressed by the classical Hall–Petch relationship, which describes the increase in the yield strength of polycrystalline metals as the grain size decreases. The particle size can reach 40 nm [50,51]. However, the plastic deformation of such materials cannot be simply defined, as it involves the interaction between dislocations and grain boundaries, which is a complex process. When the crystallites are reduced to 40 nm, they cannot accommodate multiple lattice dislocations, which involves other plastic deformation mechanisms such as grain boundary sliding, partial dislocation emission, and grain boundary absorption [52,53,54,55,56]. When the size of the crystallites is less than 20 nm, they exhibits a reverse Hall–Petch relationship, that is, the grain-boundary-assisted deformation activation causes the grain size to decrease and soften. The Hall–Petch relation and ‘‘inverse’’ Hall–Petch relation are shown in Figure 4 [57]. Although the specific plasticity mechanism of nanocrystalline metals is controversial, there is a general consensus on the grain size mechanism, dominated by dislocation-mediated mechanisms and grain boundary mechanisms. 

#### 2.2.1. Deformation Models of Polycrystals

The above-mentioned deformation of the polycrystal has two characteristics: (1) the deformation of the crystal is affected by the grain boundary and the crystal grains having different orientations; (2) the plastic deformation of any one of the grains is not in an independent, free deformation state, and the surrounding grains are required to simultaneously adapt to the deformation to maintain the bonding between the grains and the continuity of the entire object. Therefore, establishing a reasonable model to describe the plastic deformation of polycrystals has always been a hot topic for researchers. 

In the polycrystalline plastic model, the material is considered to be a composite of many preferred orientation distributions (called crystallographic texture). The plastic behavior of the metal polycrystal depends on the conversion of the microscopic physical quantity to the macroscopic physical quantity as well as on the appropriate treatment of the grain boundary effect. To describe a polycrystal, many homogenization models have been proposed based on the single-crystal plasticity theory. The most commonly used are the Sachs model [58], the Taylor model [59], and the self-consistent (SC) model [60,61].

In the 1920s, Sachs first proposed the model of polycrystal plastic deformation. This model assumes that the deformation of each grain in the polycrystal is free, that is, the stress states of the polycrystal are continuous and the same as the stress states imposed by the outside world. However, in the actual situation, the same stress everywhere will lead to a discontinuity in the strain distribution, that is, the strain will not be coordinated and will thus leads to cracks [62]. Therefore, this model is only applicable to materials with softened grains. Kroner [63] proposed the self-consistent (SC) method. The SC model is a polycrystalline averaging model that considers the interaction between grains and satisfies both geometric coordination and stress balance conditions. In the SC model, all grains are considered as an inclusion in an infinitely uniform equivalent medium (HEM).

In the 1930s, Taylor [64] proposed a model for calculating the polymorphic uniaxial stress–strain relationship, which considers polycrystals as agglomerates of randomly oriented single crystals, assuming rigid plasticity. Taylor assumed that the plastic strain is uniform and independent of the direction of the grains, i.e., the strain in any grain is equal to the average strain. The same assumptions were made about the strain rate of the grains, and the assumption that there is uniform strain in all grains means that there is a stress discontinuity at the grain boundaries. In Taylor model polycrystals are regarded as agglomerates of rigid plastic single-crystals as in Sachs model, but instead of prescribing the same stress in all the constituents, Taylor proposed to let all the grains be prescribed the same strain. The Taylor model forces the plastic strain of each grain to be the same as the macroscopic deformation, ignoring the interaction between the grains, but this does not affect its wide application. The Taylor model was successfully used to predict the texture of large deformations. Based on the Sachs and Taylor models, many improved models have been proposed. In the 1990s, Ahzi et al. [65] applied the Sachs model to large viscoplastic and elastic viscoplastic deformation. Lin et al. [66] extended the Taylor model to elastoplastic deformation, considered elastic strain, and assumed that the strain tensor of each grain is equal to the macroscopic strain rate tensor of the polycrystalline aggregate and that the critical shear stress of each slip system is the same for each grain. Referred to as the Taylor–Lin model, it is a deterministic function of cumulative shear strain. Asaro et al. [67] applied Taylor–Lin model to finite elastic viscoplastic deformation. Leffers [58] proposed an improved Sachs model instead of the Taylor model to describe the case of non-uniform multiple slip and material continuity is achieved by establishing microscopic compatibility through secondary slip. Mao et al. [59] proposed a simple and time-saving polycrystalline aggregate rolling reaction stress model based on Taylor’s principle. In this model, elastic isotropic grains deform under the action of the internal and external stress tensors caused by mechanical interaction between particles and activate the slip through the grain and the boundary regions. 

Both the Sachs model and the Taylor model ignore the actual heterogeneity that occurs during polymorphic deformation, i.e., they do not consider intergranular interactions between the grains. Takajo et al. [68] used the viscoplastic self-consistent (VPSC) polymorphic model to treat each grain as an ellipsoid inclusion, interacting with a homogeneous effective medium represented by the polycrystal, and successfully explained the mechanical response and crystal texture development during plastic deformation. The VPSC model is particularly suitable for modeling low-symmetry materials such as Mg alloys and uranium, which is a problem that cannot be solved using the traditional Taylor model.

Evidently, the above-mentioned various polycrystalline plastic models have their respective scopes of application, and they still cannot completely simulate and explain the deformation behavior and texture evolution of all grains under different loading deformation modes. At present, there are several noteworthy problems in the various models. First, certain aspects of the crystal’s constitutive relationship are not clear enough, such as work-hardening relationship, strain rate sensitivity, temperature influence, slip mechanism, and selection criteria of twinning system. The second is the interaction between grains, such as the role of grain size, stress balance between grains, and strain coordination. Therefore, further in-depth study of the crystal plastic constitutive model of the material is still needed.

**Figure 5:**
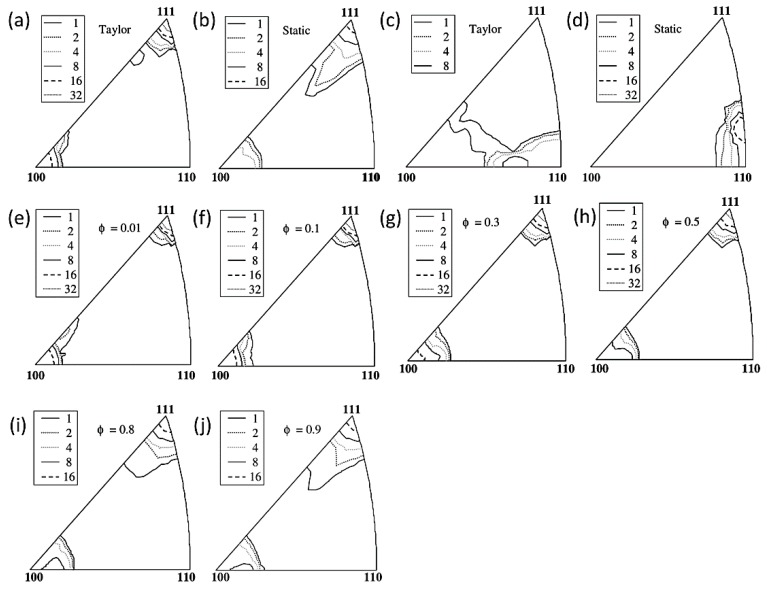
(**a**,**b**) Inverse pole figures in the case of Taylor (φ = 0) and Static models (φ = 1) at strain ε = 100% for tensile test; (**c**,**d**) inverse pole figures in the case of Taylor (φ = 0) and Static models (φ = 1) at strain ε = 100% for compression test; (**e**–**j**) inverse pole figures for tensile test at ε = 100% for the φ model (texture evolution for φ = 0.01 close to Taylor, 0.1, 0.3, 0.5, 0.8, and 0.9 close to Static for a strain level of 100% for axisymmetric tensile).

Garmestani et al. [69] used the statistical continuum theory to predict the stress–strain response and texture evolution of a polycrystalline material. This model is used to successfully predict the typical deformation texture of catalytic cracking polycrystals. Ahzi et al. [70] proposed a *φ* model for the viscoplastic large deformation of polycrystalline materials based on a new nonlinear intermediate method. The method was applied to predict the stress–strain response and texture evolution of face-centered cubic metals under tensile, compressive, and plane strain compression. In addition, the effects of weight parameters on sliding activity, stress–strain response, and texture transitions such as copper and brass textures were also analyzed (Figure 5). The proposed nonlinear rigid-viscoplastic intermediate mode model can predict the texture transition and stress-strain behavior from the upper limit (Taylor) to the lower limit (Static) by simply changing the value of the scalar weight parameter. From φ = 0.01 to 0.1, the texture of static models more obvious than Taylor model. The results obtained by the φ model are more generalized than the results obtained by the simple linear combination of the Taylor model and the static model.

#### 2.2.2. Deformation of Thin Films

A thin film is formed by different deposition techniques on the surface of a substrate (metal or nonmetal) to form a cover layer having a certain thickness (less than 10 microns) and strengthening, protection, or special function. According to its mechanical properties, a thin film can be categorized as a brittle or ductile film. There are many factors affecting the mechanical properties of the film material. The elastic modulus and stress–strain relationship of the film affect the stress change of the film material after deformation, and the strength factor determines whether the film is damaged during use. The mechanical properties of the film material directly determine the range and lifetime of the thin-film material.

In the past ten years, nano-films have played a significant role in the development of nano/microelectronic devices owing to their high strength, wear resistance, and good resistance to radiation [71,72,73]. The elastic modulus, hardness, and plastic deformation of nano-film materials are different from those of traditional thin-film materials. Nano-films exhibit superelastic modulus, toughness, and plasticity, and at nanometer scale, their mechanical properties are related to size [74,75,76]. In general, the size of the material includes geometrical dimensions such as film thickness or sample size and the grain size and other microstructure dimensions inside the material. According to previous studies, when the grain size of the material is reduced to the nanometer scale, the occurrence and movement of dislocations inside the grain are strongly restricted. The coordinated deformation of the interface, such as grain boundary slip and grain rotation, are more likely to occur. For multilayer film materials, these interfaces also strongly restrict dislocations owing to their not only ultrafine or even nano-sized grains but also a large number of layer/layer heterointerfaces. 

**Figure 6:**
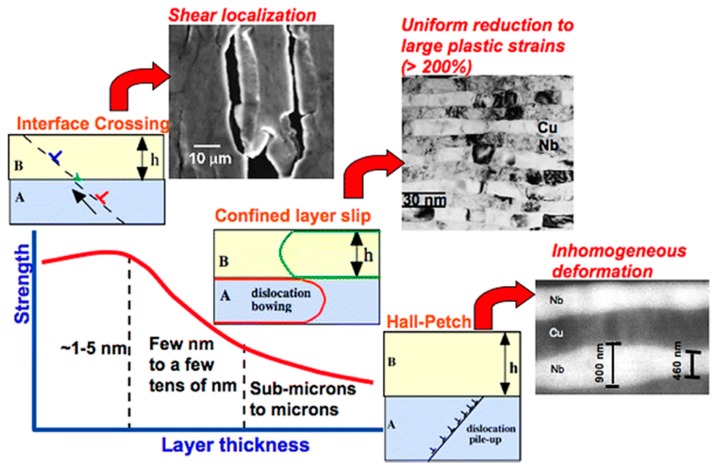
Schematic of the deformation mechanisms in metallic multilayers as a function of layer thickness from micrometer to nanometer range (The Hall–Petch mechanism of dislocation stacking can explain the relationship between layer thickness and strength at the submicron to micrometer scale. When the layer thickness is from a few nanometers to a few tens of nanometers, the constraining layer slip stress exceeds the interface slip transfer barrier, resulting in interface shear and strain localization, which limits uniform deformation).

Figure 6 shows the deformation mechanism of a metal multilayer material as a function of layer thickness from micron to nanometer [77]. At the submicron to micron scale, the Hall–Petch mechanism of dislocation stacking can be used to explain the relationship between layer thickness and strength. Dislocation stacking deformation results in uneven slip distribution and uneven layer thickness reduction. When the layer thickness is from a few nanometers to a few tens of nanometers, since there are single dislocations confined layer slip in both layers, the layer thickness is uniformly reduced, and no dislocation cell structure is formed, the initial crystal orientation relationship between Cu and Nb is retained. At layer thickness of 1–5 nanometers, the constraining layer slip stress exceeds the interface slip transfer barrier, resulting in interface shear and strain localization, which limits uniform deformation. The ability to deform a material at several nanometer scales can be achieved by making a bimodal multilayer structure in which several nanometer-thick layers alternate with tens of nanometer-thick layers.

In the grinding process of thin-film materials, it is necessary to simultaneously remove thin layers with very different properties. Under the action of mechanical load, burrs on the soft layer, debris on the brittle layer, and interfacial delamination will inevitably occur. This surface damage and structural failure can have a significant negative impact on the connection between the two thin-film modules, ultimately affecting the performance of the device [78]. A variety of techniques have been developed to characterize the destruction of thin-film structures, such as tape stripping test [79], peel test [80], and three-point/four-point bending test [81]. However, owing to the large differences in sample requirements and test environments, the results of different test methods for the same sample may be inconsistent. Nanoindentation and nanoscratch are widely used to characterize the mechanical properties of small-scale homogenous materials and the adhesion of thin films [82].

In the past two decades, the structural failure of thin-film systems has been extensively studied. Delamination at interfaces is a common form of failure in the processing of thin-film structures [83,84]. Studies have shown that the accumulation of film/substrate interfacial tension during unloading is the main cause of indentation delamination. In addition, the impact of stress evolution during abrasive processing has also been studied. The finite element method (FEM) is an effective method for analyzing interface stratification [85,86]. Understanding the interface stress distribution will provide an intuitive way to reveal the potential failure mechanism of thin-film systems in grinding. 

**Figure 7:**
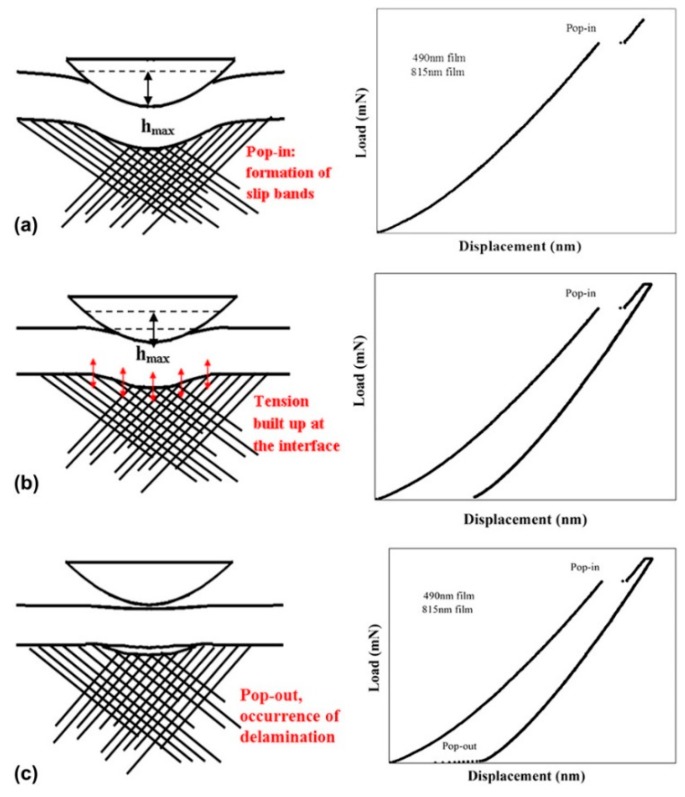
Schematic illustration of deformation mechanisms in metallic multilayers as a function of layer thickness from micrometer to nanometer range. (**a**) During the loading process, both the film and the substrate are deformed. When the stress on the substrate reaches the threshold, the substrate begins to produce a slip band, resulting in “pop-in”. (**b**) During the unloading process, both the film and the substrate are elastically restored, and a phase tension is formed at the interface due to the elastic mismatch between the film and the substrate and the difference in the existing plastic deformation; (**c**) When the tensile stress exceeds the interface strength, delamination occurs, and the peeling film elastically recovers upward during the unloading process. This speeds up the tip extraction, causing the unloading curve to “pop-out”.

Lu et al. [87] deposited a layer of amorphous silicon nitride film on a gallium arsenide (GaAs) (001) substrate using a conical indenter and nanoindentation. As shown in Figure 7, When the indentation load exceeds the critical value, the “pop-in” and “pop-out” can be observed from the load–displacement curve. The pop-in that occurs during the loading process is related to the slip of the GaAs substrate, and the pop-out that occurs during the unloading process is related to the interfacial delamination of the film and the substrate. The FEM is used to analyze the stress evolution during loading. The results of finite element analysis show that during the drawing process, the interfacial stress changes from compressive stress to tensile stress, and interface debonding is induced under critical tensile stress.

**Figure 8:**
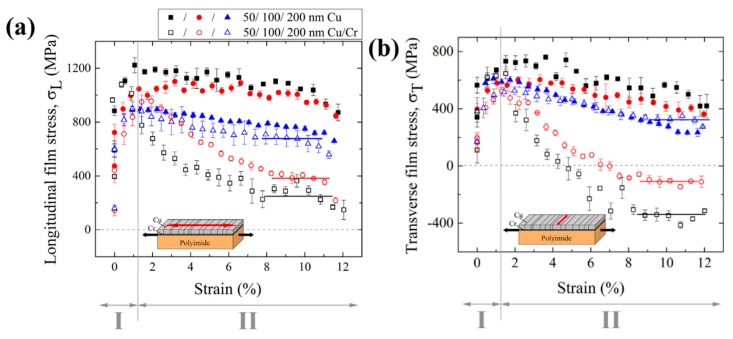
(**a**) Longitudinal film stresses, σ_L_, and (**b**) the transverse film stresses, σ_T_, in Cu films and in Cu layers of the Cu/Cr films with 50, 100, and 200 nm Cu layer thicknesses. The film stresses measured with the sin ^2^φ method are plotted against the engineering strain, ε, determined by the tensile device. The results for Cu films are marked with full symbols and for the Cu/Cr films with open symbols. The first points of the curves correspond to the residual stresses measured before the tensile test. The inset diagrams show the tensile direction (black arrows) and the measuring direction of the film stresses (red arrow). The standard deviation is used to calculate the error bars. In order to guide the eye, the solid lines indicate the plateaus of the crack saturation region in the Cu/Cr films.

Flexible electronic devices typically deposit a thin metal film on a polymer substrate. However, delamination of a conductive metal film (usually copper or silver) from the substrate may result in the loss of the electrical function. To improve the bonding strength between the metal film and the substrate, different brittle intermediate layers such as Cr, Ti, or Ta [88,89,90] may be used. Marx et al. [91] studied the effect of the brittle chromium layer on the deformation behavior of copper films on flexible substrates. The experimental results show that the chromium layer plays a leading role in the deformation process. At low strain, all membrane stresses of bare copper and Cu/Cr films are affected by film thickness and grain size. The peak stress increases with a decrease in film thickness (Figure 8, region I). At higher strains (within the crack saturation range), two-film systems begin to exhibit different mechanical properties (Figure 8, region II). The copper–chromium film is affected not only by the film thickness and grain size but also by the brittle chromium intermediate layer. The bare copper film still follows the trend of being smaller and stronger, and thinner the film with the smallest grain size, the higher is the film stress. In general, all brittle intercalations that promote bonding, such as in Cr, Ti, or Ta, can cause damage to the adherent film under mechanical loading. Moreover, the FEM explains the behavior of the Cu/Cr film. The formation of cracks is the cause of decrease in average longitudinal stress, and the plasticity of the copper layer plays a significant role in this process.

## 3. Conclusions

In recent years, nanomaterials have been used in increasing number of fields. Therefore, research on the preparation process and deformation mechanism of nanomaterials has received extensive attention. The irregular arrangement of atoms on the grain boundaries makes the energy stored inside the material high. Therefore, the grain boundaries become ready sources of defects. On one hand, under the energy drive, the grain size will gradually grow to reduce the area of the grain boundary; on the other hand, the size, distribution, and deformation mode of the grain are closely related to the material properties, service life, and different grains sizes. However, when studying the plastic deformation of materials, the classical model does not take into account the changes in the internal microstructure of the material and the unevenness of the deformation. The deformation phenomenon cannot be well explained. Therefore, studying the deformation mechanism of metal materials is of great significance for better study of the mechanical properties of metal materials. With the diversification of the mechanical properties of materials, existing material models have been unable to provide a reasonable description. The FEM is an effective calculation method for calculating the plastic deformation of materials. It can reflect the anisotropic properties of materials well and has important guiding significance for studying the deformation unevenness of materials and the evolution of texture. With the continuous development of characterization methods and calculation methods, the understanding of the interaction of grain boundary/twist boundary, dislocation, crack, mechanical twinning and shear band in nanostructured metal materials will continue to deepen. The law of strength and toughness of metal materials can be theoretically revealed, which provides a theoretical basis for the preparation of nanomaterials and their engineering applications. 

## Figures and Tables

**Table 1:** Critical resolved shear stresses of some metal single crystals with different crystal structures at room temperature.

Metal	Crystal Structure	Purity (%)	Slip Plane	Slip Direction	CRSS (MPa)
Zn	HCP	99.99	(0001)	[1120]	0.18
Mg	HCP	99.99	(0001)	[1120]	0.77
Ag	FCC	99.99	(111)	[110]	0.48
		99.97	(111)	[110]	0.73
		99.93	(111)	[110]	1.3
Cu	FCC	99.99	(111)	[110]	0.65
		99.98	(111)	[110]	0.94
Fe	BCC	99.96	(110)	[111]	27.5
			(112)		
			(123)		

**Table 2:** The largest Schmid factors on the dislocation slip and deformation twinning systems for the four loading orientations tested in body-centered cubic (BCC) W nanowires.

Loading Orientation	Dislocation		Twinning		Dominant Mechanism in Experiment
	Slip System	Schmid Factor	Twin System	Schmid Factor	
[$\bar{1}$10]	1/2[1$\bar{1}$1]($\bar{1}$01)	0.41	1/6[1$\bar{1}$1]($\bar{1}$12)	0.47	Twinning
[112]	1/2[1$\bar{1}$$\bar{1}$](101)	0.41	1/6[1$\bar{1}$$\bar{1}$](211)	0.39	Dislocation slip
[100]	1/2[11$\bar{1}$](1$\bar{1}$0)	0.41	1/6 [11$\bar{1}$](2$\bar{1}$1)	0.47	Twinning
[$\bar{1}$11]	1/2[1$\bar{1}$1]($\bar{1}$01)	0.27	1/6[1$\bar{1}$1]($\bar{1}$12)	0.31	Twinning
